# Dll4-Notch Signalling Blockade Synergizes Combined Ultrasound-Stimulated Microbubble and Radiation Therapy in Human Colon Cancer Xenografts

**DOI:** 10.1371/journal.pone.0093888

**Published:** 2014-04-15

**Authors:** Ahmed El Kaffas, Joris Nofiele, Anoja Giles, Song Cho, Stanley K. Liu, Gregory J. Czarnota

**Affiliations:** 1 Department of Radiation Oncology, Sunnybrook Health Sciences Centre, Toronto, Ontario, Canada; 2 Imaging Research and Physical Sciences, Sunnybrook Health Sciences Centre, Toronto, Ontario, Canada; 3 Departments of Medical Biophysics, University of Toronto, Toronto, Ontario, Canada; 4 Department of Oncology Research, MedImmune, Maryland, United States of America; Medical College of Wisconsin, United States of America

## Abstract

Tumour vasculature acts as an essential lifeline for tumour progression and facilitates metastatic spread. Novel vascular targeting strategies aiming to sustain vascular shutdown could potentially induce substantial damage, resulting in a significant tumour growth delay. We investigated the combination of two novel complementary vascular targeting agents with radiation therapy in a strategy aiming to sustain vascular disruption. Experiments were carried out with delta-like ligand 4 (Dll4) blockade (angiogenesis deregulator) treatment administered in combination with a radiation-based vascular destruction treatment in a highly aggressive well-perfused colon cancer tumour line implanted in female athymic nude mice. Tumours were treated with permutations of radiation, ultrasound-stimulated microbubbles (USMB) and Dll4 monoclonal antibody (mAb). Tumour vascular response was assessed with three-dimensional power Doppler ultrasound to measure active flow and immunohistochemistry. Tumour response was assessed with histochemical assays and longitudinal measurements of tumour volume. Our results suggest a significant tumour response in animals treated with USMB combined with radiation, and Dll4 mAb, leading to a synergistic tumour growth delay of up to 24 days. This is likely linked to rapid cell death within the tumour and a sustained tumour vascular shutdown. We conclude that the triple combination treatments cause a vascular shutdown followed by a sustained inhibition of angiogenesis and tumour cell death, leading to a rapid tumour vascular-based ‘collapse’ and a significant tumour growth delay.

## Introduction

Tumours require activation of an angiogenic ‘switch’ to grow beyond 1–2 mm^3^ in volume. Vascular development in tumours occurs via the release of various endothelial growth factors and angiogenesis regulators (i.e. vascular endothelial growth factor (VEGF), platelet derived growth factor (PDGF), delta-like ligand 4 (Dll4)), leading to neo-vascularization predominantly via a sprouting process reminiscent of embryonic development [1]–[3]. Newly formed vessels provide a lifeline of nutrients and oxygen to permit further tumour growth. Anti-angiogenic drugs, originally designed to block the development of tumour vasculature, have become important cancer therapies. For some time, pharmacological anti-VEGF agents gained important ground in targeting angiogenesis, but were found to be less effective than originally anticipated due to tumour resistance mechanisms [4], [5]. However, these agents were shown to be useful when strategically combined with other anti-cancer therapies [6]. Presently, pharmacological agents targeting different aspects of tumour angiogenesis are being developed and investigated in conjunction with various existing therapeutic modalities.

A variety of physiological and biological factors stimulate tumour vascular development (i.e. hypoxia). These stimuli act as external queues for angiogenic (i.e. VEGF, Dll4) molecular signalling. The Notch pathway has been demonstrated to regulate the sprouting of new vessels in tumours [7], [8]. The Notch ligand Dll4 was found to provide critical downstream regulatory signalling for initiating VEGF stimuli [9], [10]. This pathway has been identified as a promising new cancer treatment target [9]. Studies have demonstrated that inhibiting Dll4 signalling results in excessive but non-functional angiogenesis in tumour tissue [11], [12]. In addition, Dll4 blockade causes a significant tumour growth delay in various tumour cell lines [11], [12]. Studies have also demonstrated the potential of Dll4 blockade in combination with other cancer therapies [8], [11]–[15]. A recent study observed synergistic enhancement of tumour growth delay when a neutralizing Dll4 monoclonal antibody (mAb) was combined with radiation therapy [15].

Alongside the numerous pharmacological agents that have been developed to target angiogenesis, emerging biophysical vascular targeting agents, including ultrasound-stimulated microbubbles and gold nanoparticles, have also been demonstrated to increase conventional cancer treatment efficacy [16]–[19]. These have been suggested to cause a rapid onset of tumour vascular shutdown and/or increased vessel wall permeability, acting to strategically enhance the efficacy of cancer drugs or radiation therapy. More recently, Czarnota *et al.* [19]–[23] demonstrated that ultrasound-stimulated microbubbles (USMB) enhances tumour cell death in prostate, bladder and breast cancer cell lines. They posited that enhancement of radiation response was linked to the biophysical membrane damage of tumour endothelial cells associated with microbubble stimulation, subsequently leading to ceramide signalling and a vascular shutdown due to endothelial cell death [22]. The observations are potentially linked to recently reported, *asmase*-dependent, acute endothelial apoptosis following single large doses of radiation [2], [24], [25].

In this work, we have investigated Dll4-blockade following combined ultrasound-microbubble and radiation-based vascular destruction in a highly aggressive and well-perfused colon cancer line. Tumours were grown in the hind leg of mice and treated with permutations of radiation, USMB and Dll4 monoclonal antibody (mAb). Animals were imaged using three-dimensional power Doppler ultrasound to investigate changes in tumour perfusion (blood flow) with treatment.

Three-dimensional high-frequency power Doppler ultrasound is an ideal imaging modality for the pre-clinical assessment of tumour vasculature in animal models [26]–[28]. It yields high-resolution structural information and enhanced Doppler flow signal detection. In addition, its facile use makes it suitable for longitudinal studies, where relative changes in vasculature can be assessed. Doppler results were complemented with vascular densities determined from CD31 staining of tumour cross-sections. Tumour response to Dll4 mAb, ultrasound-microbubbles and radiation combination therapy was also assessed with measurements of immunohistochemistry and tumour growth assays.

The results indicate that Dll4 blockade followed by USMB and radiation produces a synergistic tumour growth delay greater than when radiation is combined with Dll4 mAb alone. These findings also suggest that USMB and radiation act to acutely shut down tumour vasculature, as in other types of tumours, but that due to the aggressive nature of the colon cancer cell line, a rapid tumour rebound follows. Treatment with Dll4 mAb then deregulates neo-vascularization, leading to minimally functional tumour vasculature and a sustained tumour vascular ‘collapse’. Thus, the combination of DLL4 blockade with USMB and radiation is a novel, highly effective approach to delay tumour growth through biological and biophysical targeting of the tumour vasculature.

## Materials and Methods

### Xenograft Tumour Model

All animal experiments presented in this work were conducted in compliance with internationally recognized guidelines specified in protocols approved by the Sunnybrook Research Institute institutional animal care and use committee. All animal procedures were performed under anaesthesia, as described below, and all efforts were made to minimize suffering. For this study, up to 90 animals were randomly divided into 3 cohorts. Animals in cohort 1 were sacrificed at 24 hours, animals in cohort 2 were sacrificed at 7 days and animals in cohort 3 were kept for tumour growth assessment for up to 30 days. In order to initiate tumour growth, LS174T (3×10^6^) colon cancer cells (ATCC, Manassas, VA) were injected subcutaneously into the right hind leg of 6 to 7-week-old female athymic nude mice (Harlan, Indianapolis, IN). All tumours grew for 9–14 days to reach a mean volume of 200 mm^3^. An average of 5 animals were used per treatment condition for each of the treatment assessment methods (i.e. power Doppler, growth delay, immunohistochemistry).

### Treatment Administration

Tumour-bearing animals were given one of six treatments: (1) no treatment (CTRL), (2) radiation (XRT), (3) Dll4 mAb, (4) XRT and Dll4 mAb, (5) ultrasound-stimulated microbubbles (USMB) combined with XRT, or (6) a triple combination of USMB, XRT and Dll4 mAb. Animals were anesthetised with isofluorane before imaging or for catheter insertion (when receiving USMB treatment). All animals receiving radiation therapy were treated with a single dose of 5 Gy ionizing radiation administered using a Faxitron cabinet irradiator (Faxitron Bioptics, Lincolnshire, IL), using X-rays at an energy of 160 kVp, an SSD of 35 cm, and a dose rate of 200 cGy/min. For radiation treatment, the body of each animal was covered with 3 mm thick lead shielding, exposing only the tumour. Animals receiving the Dll4 monoclonal antibody (mAb; MedImmune LLC, MD) were administered the mAb twice weekly, starting on the treatment day following imaging, at a dose of 5 mg/kg of body weight. Animals receiving radiation in conjunction with Dll4 mAb, received the initial anti-Dll4 mAb treatment 1 hour after irradiation. Dll4 mAb treatment was continued in animals until sacrifice (at 24 hours or 7 days), or until a tumour volume reached at least double the original volume. A schematic of the treatment delivery and imaging schedule is presented in figure S1A.

For animals treated with USMB, Definity microbubbles (perfluoropropane gas/liposome shell, Lantheus Medical Imaging, N. Billerica, MA) were prepared using a Vialmix device (Lantheus Medical Imaging) and administered through a tail vein catheter at a volume concentration of 3% v/v (1.08×10^9^ microbubbles in 90 µL), which is approximately 300-fold the recommended diagnostic imaging dose of 10 ul/kg [19]. Animals were then immersed in a water bath at 37°C in order to be exposed to ultrasound. The treatment system is designed in such a way that only the lower body of the animal is immersed in the water bath, exposing only the tumour volume to the ultrasound field at the focus of the transducer. The ultrasound therapy system involved a micro-positioning system, waveform generator (AWG520, Tektronix, Beaverton, OR), power amplifier with pulser/receiver (RPR4000, Ritec, Rochester, NY), and a digital acquisition system (CC103, Agilent, Monroe, NY). Tumours were exposed within the half maximum peak of the acoustic signal (−6 dB beam width at focal point of 3.1 cm, and focused at 8.5 cm) using a 16-cycles tone burst at 500 kHz center frequency with a 2.86 cm element diameter ultrasound transducer (ILO509HP, Valpey Fisher Inc., Hopkinton, MA). A 3 kHz pulse repetition frequency over 50 ms was used for sonification, along with a peak negative pressure set to 570 kPa, corresponding to a mechanical index (MI) of 0.8. Total sonification time was ∼ 5 ms within each 50 ms window. An intermittent 1950 ms period between sonification windows was employed to minimize biological heating in the tissue during ultrasound exposures. The total tumour insonification time was 750 ms over 5 minutes for an overall duty cycle of 0.25% [19]. This particular pulse sequence has been demonstrated to have good radiosensitizing effects, while enabling blood vessels to refill with bubbles 150 times during treatment. Tumours were irradiated with a 5 Gy dose of ionizing radiation immediately after USMB treatment.

### High-Frequency Ultrasound Power Doppler Imaging

All animals were imaged with three-dimensional high-frequency ultrasound immediately before treatment and 24 hours after treatment. Data was acquired using a Vevo 770 high-frequency ultrasound-imaging device (Visualsonics, Toronto, ON) with a 30 MHz center frequency transducer (RMV-707∶55 µm axial resolution, 115 µm lateral resolution, focal length of 12.7 mm). A motorized scan stage (Visualsonics, Toronto, ON) was used to acquire 3D B-mode images and power Doppler flow data, at a step size of 0.2 mm. Power Doppler data was collected with the following settings: a clutter-filter cut-off of 1–2 mm/s, a scan speed of 1 mm/s, a pulse repetition frequency of 5 KHz, a power Doppler gain of 20 dB and a frame rate lesser than 10 fps. Power Doppler image analysis was conducted using in-house developed software in MATLAB (The MathWorks, Natick, MA). The vascularity index (VI) was computed from power Doppler images by obtaining the volume of all coloured objects (coloured pixels) over the volume of the selected ROIs. A relative vascularity index was used to assess overall vascular response to therapy, computed as follows: ((VI_24 Hours_ **/**VI_0 Hours_)×100) –100) or ((VI_7 Days_ **/**VI_0 Hours_) ×100) –100).

### Histological Analysis

Mice were sacrificed 24 hours, and in another cohort, at 7 days after treatment for histology and immunohistochemistry. Cross sections of tumour xenografts were stained with hematoxylin & eosin (H&E) to evaluate necrosis following therapy. Tumour xenograft cross-sections were also stained for DNA breaks using *in situ* end labelling (ISEL) stain as a cell death marker, and cluster of differentiation (CD31) (Santa Cruz Biotechnology, Santa Cruz, CA) to evaluate vascular density. Cell death was quantitatively evaluated for each of the tumour ISEL-stained cross-sections. This was carried-out using custom MATLAB (The MathWorks, Natick, MA) routine, which allows the user to compute the number of brown pixels (ISEL-stained) over the total number of pixels per whole tumour cross-section. The vascular density was also computed from images of CD31 stained tumour cross sections. For each cross section, 4 fields of view (FOV) were analyzed at an objective lens magnification of 10X to compute the density of vasculature. This was carried out using MATLAB (The MathWorks, Natick, MA) to distinguish the brown stained regions (CD31 staining of endothelial cells) in the tumour from the background stain (other cells).

### Tumour Growth Assessment

Tumour growth was assessed in an average of 5 animals per treatment condition. Tumour volume was determined by calliper measurements performed every 2–4 days and calculated by using the modified ellipsoidal formula (volume = length × width^2^/2). Tumour volumes were normalized to their starting volume and tumour growth delay was calculated by subtracting the average number of days for treatment groups to reach two times their starting volume from that of the control group.

### Statistical Analysis

Quantified relative VI, ISEL and CD31 staining and tumour growth delays, were evaluated for statistical significance using a Mann-Whitney test (two-tailed, assuming unequal variances; α = 0.05). Each treatment condition was compared directly to the 0 Gy control condition. In addition, p values from statistical tests of different permutations have been summarized in Tables S1 to S7 in the supplementary data. Statistical tests were conducted using Prism (GraphPad Software version 5, La Jolla, CA).

## Results

**Figure 1:**
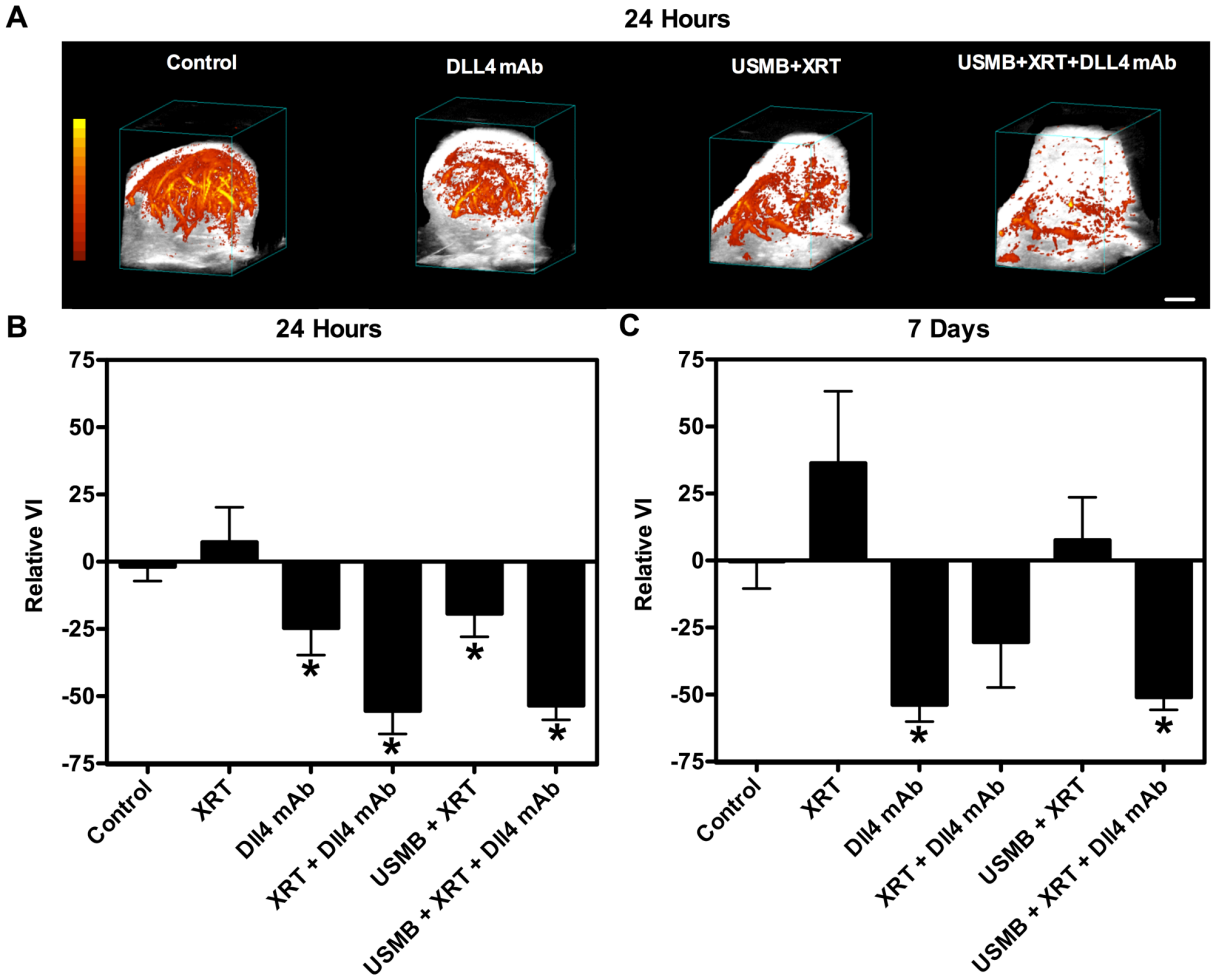
Power Doppler results. A) Representative images of three-dimensional maximum intensity projection of power Doppler signal at 24 hours post treatment, and quantified power Doppler at B) 24 hours and C) 7 days, are shown. Animals receiving Dll4 mAb alone had a decrease in flow signal (VI) 24 hours after treatment delivery; the VI dropped further 7 days after therapy. USMB+XRT caused a rapid decrease in power Doppler signal. However, the active blood volume returned to baseline levels by 7 days. A rapid VI decrease of almost 60% was observed for XRT and Dll4 mAb as well as triple combination conditions. However, the VI drop persisted to 7 days only in animals receiving the triple condition treatment. Each experimental condition is represents an average of 5 animals. Error bars represent one standard error of the mean. Statistically significant changes are marked with * (p≤0.05). The scale bar represents 2 mm.

We conducted experiments to assess the efficacy of vascular targeting strategies where USMB, radiation and anti-Dll4 treatments were combined to disrupt tumour vasculature and sustain vascular effects in order to maximize overall tumour response (Figure S1B). Tumour vasculature response was assessed with three-dimensional high-frequency power Doppler ultrasound at 24 hours and 7 days after treatment, relative to baseline. Figure 1A displays representative images of three-dimensional power Doppler data at 24 hours after treatment for the control (1), Dll4 mAb (3), USMB+XRT (5) and USMB+XRT+Dll4 mAb (6) treatment conditions. A decrease in flow signal was apparent in tumours treated with the triple combination (6). Quantified power Doppler results at 24 hours and 7 days after treatment are presented for all treatment conditions in figure 1B and 1C. Minimal changes in the VI were observed for control animals (1) at both times. We observed a non-significant increase in power Doppler signal related to blood flow at 24 hours, and 7 days following XRT (2). Animals receiving Dll4 mAb (3) alone were observed to have a decrease in flow signal (VI) 24 hours after treatment (p<0.05). In addition, treatment with Dll4 mAb alone (3) caused the VI to drop further 7 days after therapy (p<0.05). Treatments with USMB+XRT (5) also caused a rapid (within 24 hours) and significant (p<0.05) decrease in power Doppler signal. However, the blood flow volume differences by power Doppler returned to baseline levels by 7 days, indicating a temporary, short-lived microbubble-based vascular disruption. A rapid VI decrease of almost 60% was observed for treatments with XRT and Dll4 mAb (4), as well as the triple combination conditions (6) (p<0.05). However, the VI drop persisted to 7 days only in animals receiving the triple combination (6) treatments or those receiving Dll4 alone (3) (p<0.05). In contrast, an increase of ∼ 25% in the VI for XRT+Dll4 mAb (4) treated animals indicates a potential reperfusion of the tumour.

**Figure 2:**
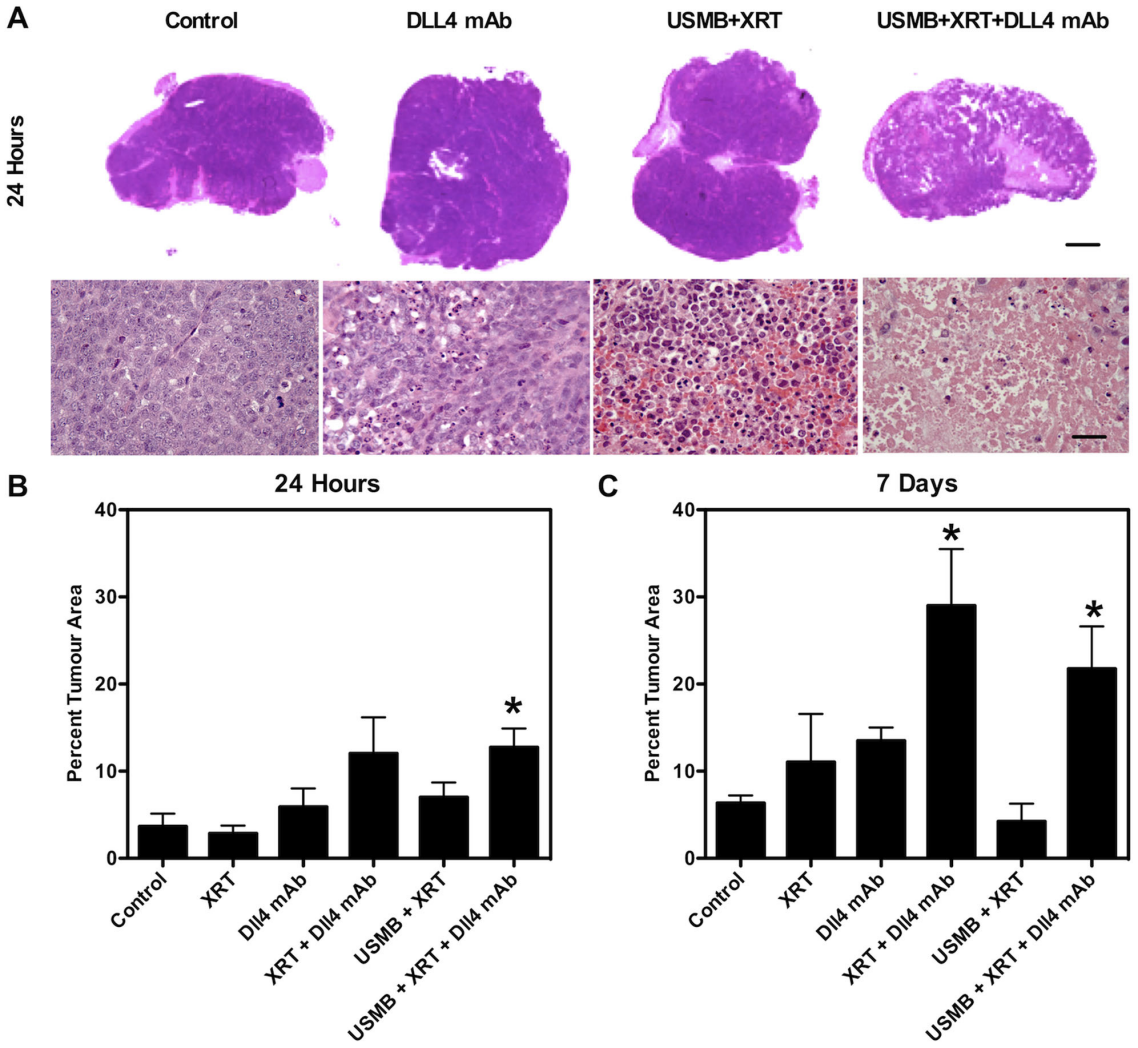
Cell death results. A) Representative 24 hour H&E stained tumour cross-sections for specified treatment conditions at low magnification. The bottom row is of high-magnification images of representative H&E stained tumour cross-sections for the specified treatments. The top row scale bar represents 2 mm and the bottom row scale bar represents 50 µm. Quantified ISEL staining at B) 24 hours and C) 7 days. Significant (p≤0.05) cell death was observed in tumours treated with USMB+XRT+Dll4 mAb, while a non-significant amount of cell death is observed for animals treated with XRT and Dll4 mAb. At 7 days after initial treatment, we noted significant (p≤0.05) amounts of cell death in both the combined XRT+Dll4 mAb and USMB+XRT+Dll4 mAb treatment conditions. Statistical significance is indicated with * for p≤0.05.

Figures 2A, S2A and S2B display representative H&E and ISEL stained tumour cross-sections for each of the treatments. Data demonstrate cell death at 24 hours in the combined XRT and Dll4 mAb (4) treatment, as well as the triple combination (6) treatment condition. Cell death regions persisted in tumours at 7 days after treatment in both these conditions. Quantified cell death areas from ISEL stained tumour cross-sections are presented in Figures 2A and 2B. Significant (p<0.05) cell death was observed in tumours treated with USMB+XRT+Dll4 mAb (6), while a non-significant increase in cell death was observed for animals treated with radiation combined with Dll4 mAb (3). At 7 days after initial treatment, we noted significant (p<0.05) amounts of cell death in both the XRT+Dll4 mAb (4) and the USMB+XRT+Dll4 mAb (6) treatment conditions. Furthermore, unique tumour H&E morphology, consisting of multiple necrotic regions and leaked red blood cells, was observed in tumours receiving the triple combination at the time of the end-point (10–30 days post therapy) sacrifice (Figure S3).

**Figure 3:**
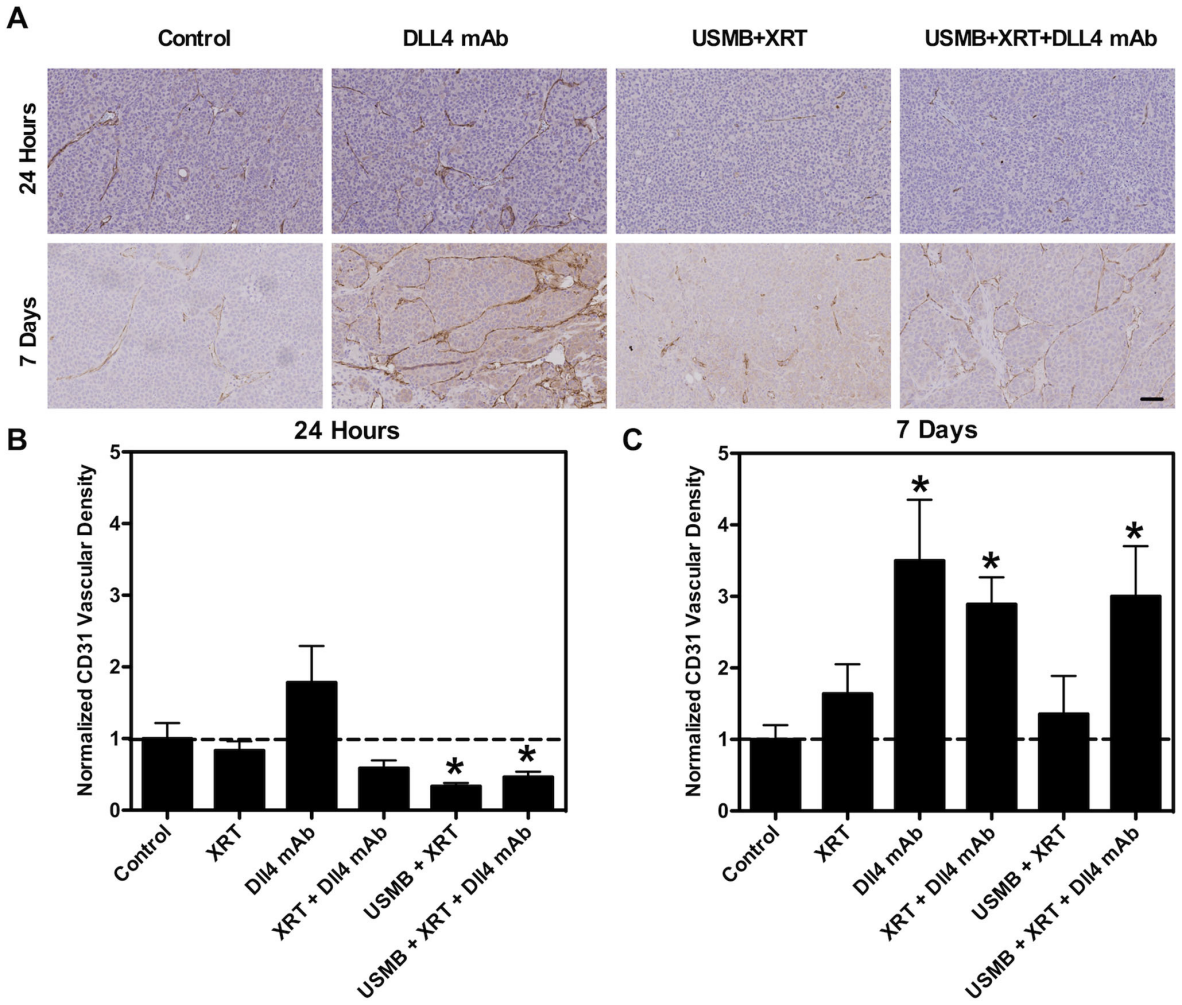
CD31 staining results. A) Representative images of CD31 staining of tumour cross-sections. Images are of four of the treatment conditions obtained at 24 hours and 7 days after therapy. The scale bar represents 100 µm. B) Normalized CD31 staining per ROI at 24 hours and C) at 7 days after treatment start. We noted a non-significant, but near doubling of CD31 staining in animals treated with Dll4 mAb only. There was a significant decrease in CD31 staining in animals treated with USMB+XRT as well as those treated with the triple treatment conditions. By 7 days after treatment, there was a significant increase in CD31 stained vessels for all animals receiving Dll4 mAb treatment, whether alone or in combination with USMB and/or XRT. Animals treated with XRT alone, or in combination with USMB, had quantified CD31 stained counts similar to control animals. Statistically significance is indicated with * indicating a p≤0.05.

Power Doppler and cell death results were complemented with results from CD31 staining. Representative CD31 immunohistochemistry is shown in figure 3A and S2C. By 24 hours after therapy (Figure 3B), we noted a non-significant, but near doubling of CD31 staining in animals treated with Dll4 mAb (3) only. Meanwhile, we observed a significant decrease in CD31 staining in animals treated with USMB+XRT (5) as well as those treated with the triple treatment conditions (p<0.05). By 7 days after treatment (Figure 3C), we observed a significant increase in CD31 staining for all animals receiving Dll4 mAb treatment, whether alone (3) or in combination with USMB and/or XRT (4 and 6). We also noted that animals treated with XRT alone (2), or in combination with USMB (5), had CD31 staining levels similar to control animals.

**Figure 4:**
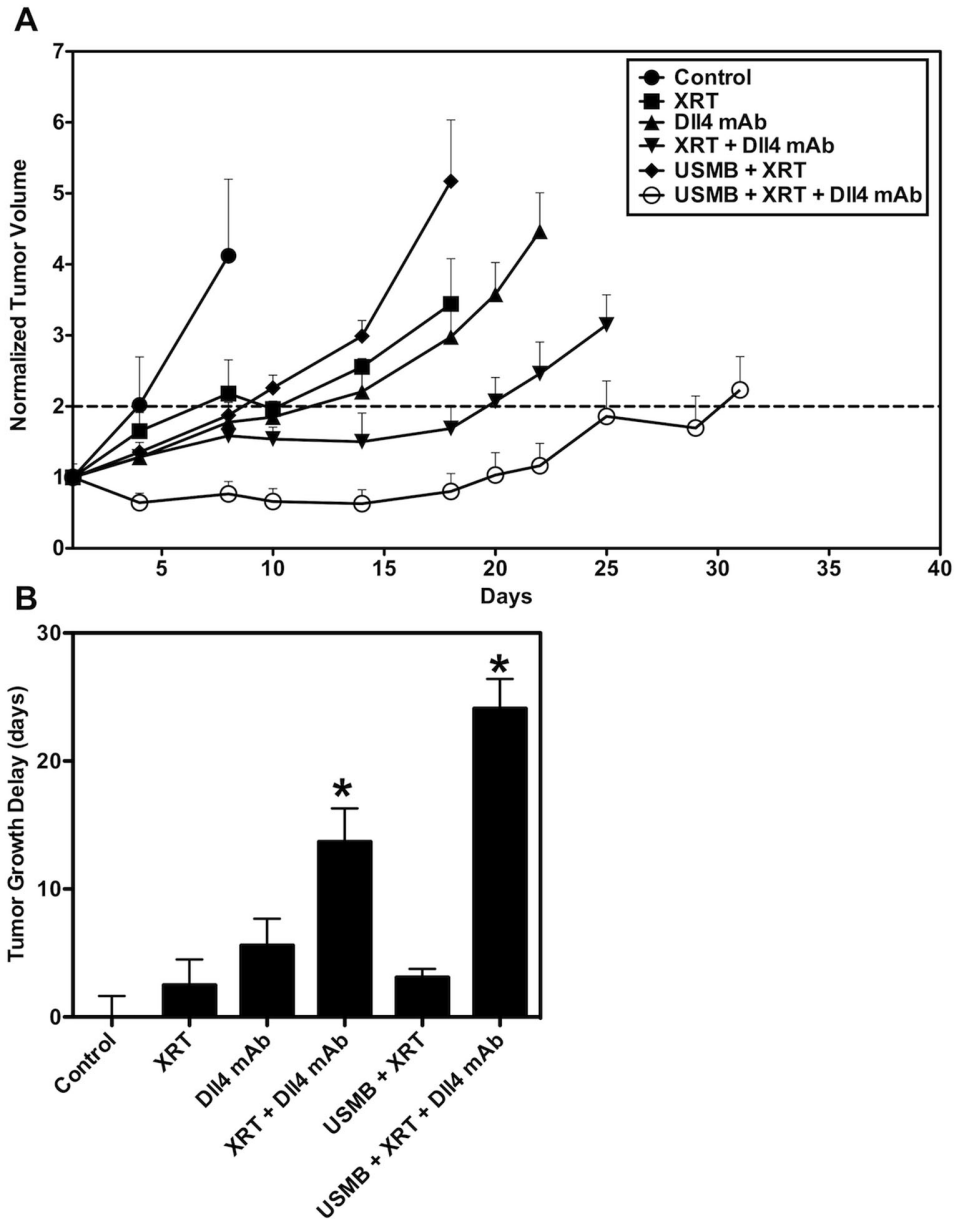
Tumour growth results. A) Normalized tumour growth curves. A delayed tumour growth in all treatment conditions was noted. Tumour growth curves followed similar trends for the XRT alone, the Dll4 mAb alone, and the USMB+XRT treatments. Combined USMB+XRT+Dll4 mAb was observed to induce the greatest response, with a decrease in tumour size at 5 days after treatment, followed by growth inhibition lasting for nearly 15 days. B) Quantified tumour growth delay to reach two times the starting volume. Treatments with XRT and continued Dll4 mAb experienced a significant synergistic tumour growth delay of 14 days. Meanwhile, the triple combination treatment (USMB+XRT+Dll4 mAb) caused a tumour growth delay of 24 days. Statistically significant differences in tumour growth delay are indicated with * for p≤0.05.

Figure 4A displays the normalized tumour growth curve for each of the treatment conditions. Whereas control animals (1) doubled in volume in less than 5 days following treatment initiation, we noted a delayed tumour growth in all other treatment conditions. Growth curves followed similar trends for the tumour growth treated with XRT alone (2), Dll4 mAb alone (3), and the USMB+XRT treatment (5). Tumours treated with XRT and continued Dll4 mAb therapy (4) demonstrated a significant and synergistic tumour growth delay (see Figure 4B) of 14 days (p<0.05). Triple combination treatments (6) caused the greatest tumour growth delay, with a slight decrease in tumour size evident at 5 days after the start of treatment, followed by an interruption in growth lasting for nearly 15 days. An average tumour growth delay of 24 days was observed (p<0.05) for the triple combination treatment.

## Discussion

**Figure 5:**
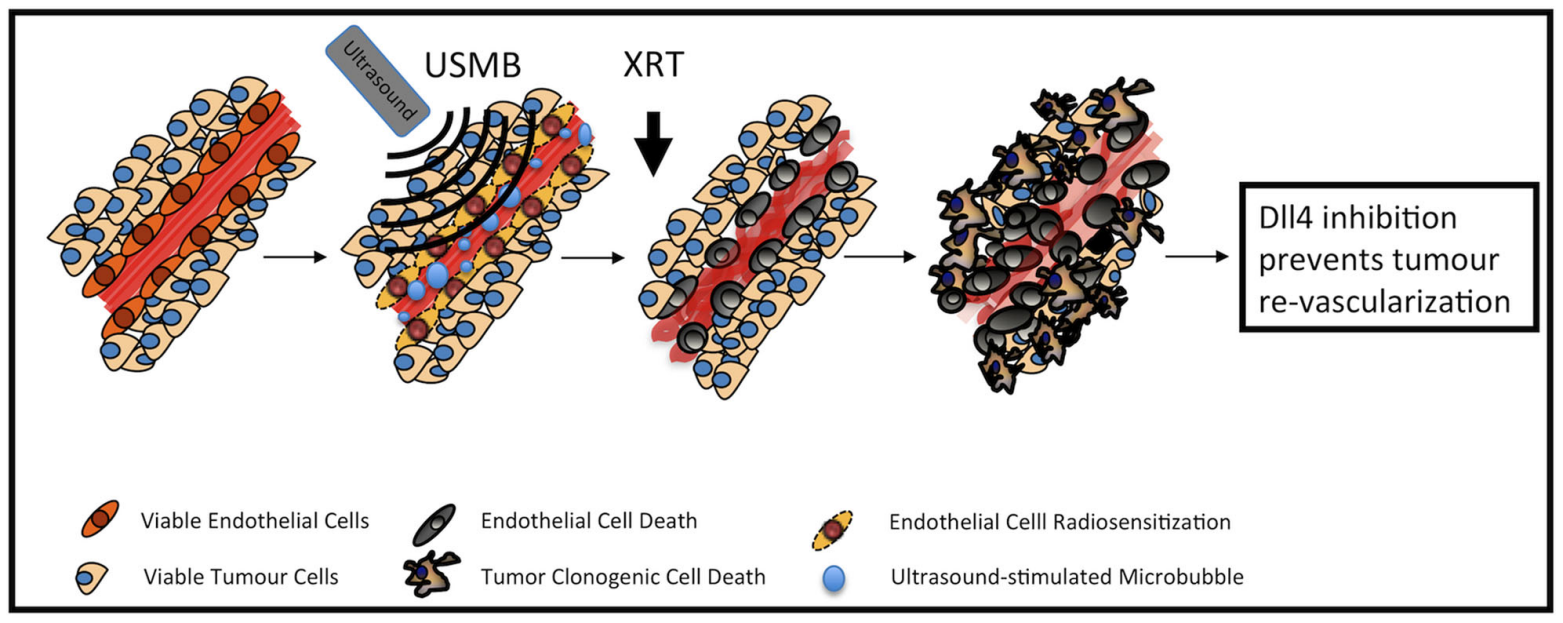
Illustration of posited USMB, XRT and Dll4 mAb effects on tumour endothelial cells. We posit that pre-treating tumours with ultrasound-activated microbubbles may first radiosensitize the cells via a biophysical process. Upon radiation delivery, endothelial cells would first undergo apoptosis, leading to tumour cell death. Continued delivery of an angiogenesis deregulator would prevent tumour reperfusion, serving as a maintenance therapy.

In this study, we set out to investigate the efficacy of strategically combining a biophysical, radiation-based, vascular disrupting treatment followed by an angiogenesis-deregulating pharmacological agent. It was posited that this would optimize treatment response. In order to maximize vascular destruction, ultrasound-stimulated microbubble therapy was used to first radiosensitize tumour endothelial cells, followed by a single dose of ionizing radiation (XRT) to induce rapid vascular effects. Dll4 mAb was subsequently administered as a ‘maintenance’ therapy. An illustration of posited combined USMB, XRT and Dll4 mAb effects on tumour endothelial cells are presented in figure 5. Different permutations of the three combined treatment modalities were also investigated. Results suggest a 1.4X synergistic tumour growth response in animals treated with one session of triple combination therapy (6), where a ∼ 24 day tumour growth delay was achieved (Figure 4). This is likely linked to a sustained tumour vascular shutdown inducing cell death within the tumour. Anti-Dll4 mAb treatment then acts to maintain vascular shutdown. In comparison, combining radiation and Dll4 mAb (4) caused a significant growth delay of ∼ 14 days. No other significance was observed in growth delay assays. Tumours receiving radiation alone experienced the least tumour growth delay (∼3 days), likely linked to conventional non-vascular radiobiological DNA damage.

A decrease in tumour size was observed within 5 days following the triple combination treatment (6), demonstrating an acute response and suggesting a rapid tumour vascular-based ‘collapse’. We observed a power Doppler VI decrease of more than 50% at 24 hours in both XRT+Dll4 mAb (4) and triple combination treatments (6), in comparison to radiation alone (2), where no decrease in the VI was observed (Figure 1). The VI decrease was only sustained to 7 days in the triple combination treatments. Tumour volume recession is likely associated with extensive vascular shutdown, leading to the rapid formation of necrotic regions in the core of the tumour. We posit that dead cells are rapidly disintegrated and cleared in tumours treated with the triple combination therapy, which would explain the less extensive quantified ISEL staining for the triple combination treatments (Figure 2), and the tumour size reduction. We have also observed a significant amount of cell death 24 hours following this triple combination therapy (6), while no other significance was found in other treatment conditions (Figure 2). Here, ISEL-quantified cell death areas per tumour cross-section persisted in the long-term (beyond 24 hours) and reached nearly a mean of 30% of the tumour cross-section by 7 days following therapy. Radiation combined with Dll4 mAb (4) also caused significant cell death 7 days following therapy, however tumour volumes in this condition were noted to be larger than tumour treated with the triple combination therapy. H&E stained images indicated a persisting heterogeneous and necrotic tumour microenvironment 30 days after therapy (Figure S3). These results clearly demonstrate the potency of combining USMB, radiation and Dll4 mAb above (6) and beyond any therapy alone.

For animals treated with XRT+Dll4 mAb (4) or USMB+XRT+Dll4 mAb (6), a significant and rapid decrease in the vascular index was observed by 24 hours following therapy (Figure 1). However, we posit that the resulting vascular shutdown in some of these treatments is of a differing nature. In USMB+XRT+Dll4 mAb (6) treated animals, endothelial cells likely undergo rapid apoptosis associated with a membrane perturbation due to microbubble mechanical effects, and resulting in ceramide production [29], [30]. Return of functional vessels in treated tumours is then supressed by Dll4 mAb treatments. In contrast, treatments with XRT+Dll4 mAb (4) are likely causing some endothelial cell death (as suggested in CD31 staining), leading to a temporary vascular shutdown [15]. At 7 days following XRT+Dll4 mAb (4) treatments, the vascular index appears to be returning to the baseline level, while the vascular index depression persists in the triple combination treated-animals.

Treatment with Dll4 mAb alone (3) induced a similar response to those previously reported [9]–[12]. As anticipated, the quantified CD31 staining increased after treatment initiation, while active blood flow, as assessed using power Doppler ultrasound, decreased (Figure 3). This is characteristic of Dll4 blockade, where excessive angiogenesis occurs, but paradoxically vascular perfusion decreases. In this study, shutdown of vascular function was sustained for up to 7 days after the start of Dll4 mAb treatment (3) (Figure 1). However, a tumour growth delay similar to radiation alone (2) was observed, indicating that Dll4 mAb based vascular shutdown is not sufficient to induce substantial tumour damage (Figure 4).

Recent investigations have demonstrated that pre-treating tumours with USMB can radiosensitize endothelial cells to radiation, subsequently increasing the overall response of tumours [19]. We have found that USMB treatment followed by XRT (5) causes an acute and significant decrease in the VI, as measured with power Doppler ultrasound (Figure 1). Quantification of CD31 staining was in agreement with this, where a significant decrease in staining was observed at 24 hours (Figure 3). However, tumour reperfusion was observed at 7 days after treatment (Figure 1). Similar observations were recently reported [23]. This effect is likely linked to the aggressive nature of the LS174T colon cancer cell line. Generally, treatment response may be a function of tumour aggressiveness and vascular architecture, an important subject of future investigations.

Following XRT, we noted an increase in the power Doppler vascularity index at 7 days (Figure 1). A similar effect was noted in animals receiving XRT+Dll4 mAb (4). Observed variations are likely linked to response variations in animals. In addition, studies have indicated that small doses of radiation can promote tumour angiogenesis, or induce inflammatory effects resulting in increased tumour perfusion [31]–[33]. It is also possible that radiation may be pruning less mature endothelial cells, leading to an enhanced vascular architecture and increased blood flow [34].

In summary, the results demonstrated an enhanced tumour response when ultrasound-stimulated microbubble therapy and XRT were combined with Dll4 mAb (6) as a maintenance therapy. Our results clearly demonstrate that a synergistic effect enhances treatment response when USMB, radiation and Dll4 mAb are strategically combined. In the future, we anticipate that a yet greater treatment response can be achieved through the administration of multiple fractionated treatments. Our results are encouraging given the increasing use of single fraction radiation therapy. Such treatments are now commonly used for palliation with the aim of eliciting rapid anti-vascular effects, in turn minimizing bleeding in patients, arresting tumour growth and alleviating pain. The use of USMB and Dll4 mAb in combination with a single radiation fraction may enhance such effects. Results presented in this study also confirm the importance of Dll4 in regulating ongoing tumour angiogenesis and its potential use in combination with other novel cancer therapies. Finally, our results add to the paradigm that tumour blood vessels play an important role in tumour response to therapy and demonstrate a maximal tumour response when tumour vasculature is biophysically disrupted, followed by an angiogenesis deregulating ‘maintenance’ treatment.

## Supporting Information

Figure S1 A) Schematic overview of experiments. Tumours were left to grow for 9–14 days. Animals were then imaged and treated accordingly. Follow up imaging took place at 24 hours and 7 days. Animals treated with Dll4 mAb continued receiving the agent until sacrifice. B) Potential model for vascular strategy. While USMB+XRT is likely causing a rapid vascular shutdown, the aggressive nature of the cell line is inducing a rapid vascular rebound. In contrast, Dll4 mAb following USMB and XRT blocks functional vascular rebounds for a longer period of time.
(TIFF)

Figure S2 **Representative A) H&E, B) ISEL, and C) CD31 stained tumour cross-section images for each of the treatment conditions at low and high magnification.** The scale bar represents 2 mm for ISEL and H&E and 100 µm for CD31.
(TIFF)

Figure S3 **Representative H&E images of control, Dll4 mAb, XRT+Dll4 mAb, USMB+XRT and USMB+XRT+Dll4 mAb at tumour growth assay end point (10–30 days, depending on treatment condition).** Persisting tumour heterogeneity is observed in the triple combination H&E stained tumour cross-section. The scale bar represents 2 mm.
(TIFF)

Table S1 **P-value summary for all quantified 24 hours ISEL staining from all treatment conditions.**
(DOCX)

Table S2 **P-value summary for all quantified 7 days ISEL staining from all treatment conditions.**
(DOCX)

Table S3 **P-value summary for all quantified growth delay from all treatment conditions.**
(DOCX)

Table S4 **P-value summary for all quantified 24 hours VI values from all treatment conditions.**
(DOCX)

Table S5 **P-value summary for all quantified 7 days VI value from all treatment conditions.**
(DOCX)

Table S6 **P-value summary for all quantified 24 hours CD31 staining from all treatment conditions.**
(DOCX)

Table S7 **P-value summary for all quantified 7 days CD31 staining from all treatment conditions.**
(DOCX)
