# Supplementary figures and images for: Dll4-Notch Signalling Blockade Synergizes Combined Ultrasound-Stimulated Microbubble and Radiation Therapy in Human Colon Cancer Xenografts
Source: PLoS One. 2014 Apr 15;9(4):e93888. doi: 10.1371/journal.pone.0093888 (PMC3988033; doi:10.1371/journal.pone.0093888)

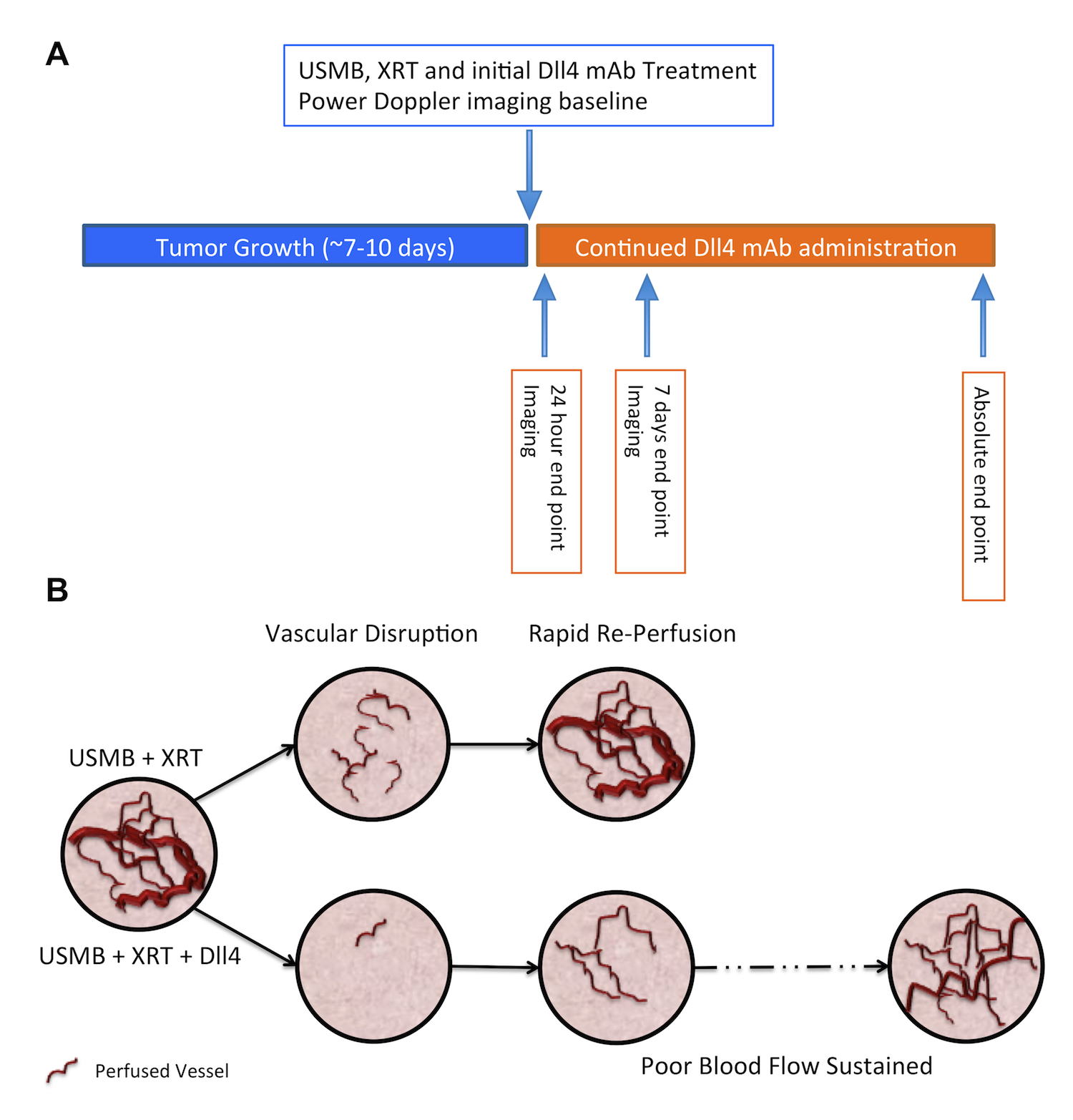

Supplement: Figure S1 — A) Schematic overview of experiments. Tumours were left to grow for 9–14 days. Animals were then imaged and treated accordingly. Follow up imaging took place at 24 hours and 7 days. Animals treated with Dll4 mAb continued receiving the agent until sacrifice. B) Potential model for vascular strategy. While USMB+XRT is likely causing a rapid vascular shutdown, the aggressive nature of the cell line is inducing a rapid vascular rebound. In contrast, Dll4 mAb following USMB and XRT blocks functional vascular rebounds for a longer period of time. (TIFF) [file pone.0093888.s001.tif]

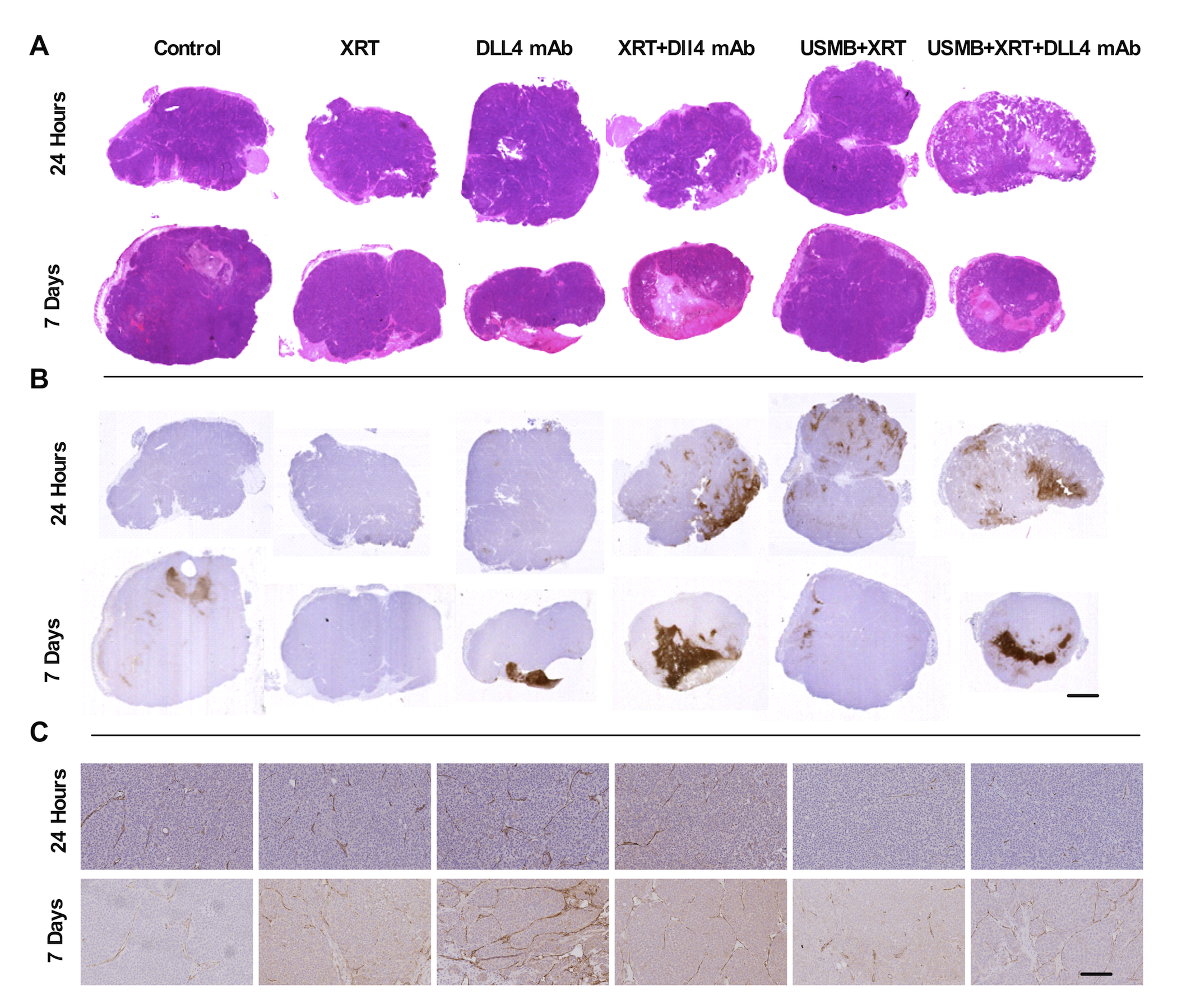

Supplement: Figure S2 — Representative A) H&E, B) ISEL, and C) CD31 stained tumour cross-section images for each of the treatment conditions at low and high magnification. The scale bar represents 2 mm for ISEL and H&E and 100 µm for CD31. (TIFF) [file pone.0093888.s002.tif]

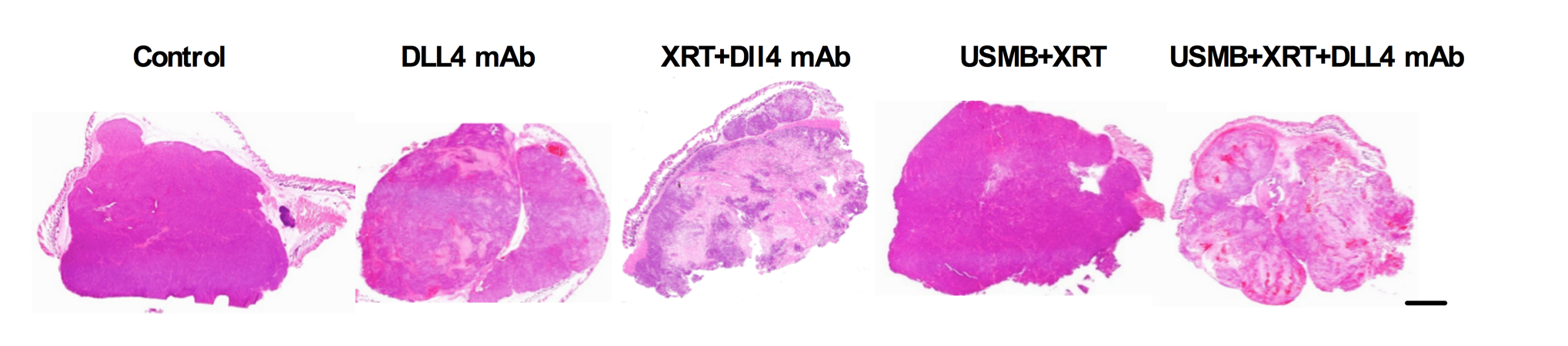

Supplement: Figure S3 — Representative H&E images of control, Dll4 mAb, XRT+Dll4 mAb, USMB+XRT and USMB+XRT+Dll4 mAb at tumour growth assay end point (10–30 days, depending on treatment condition). Persisting tumour heterogeneity is observed in the triple combination H&E stained tumour cross-section. The scale bar represents 2 mm. (TIFF) [file pone.0093888.s003.tif]
