# Supplementary material for: Dll4-Notch Signalling Blockade Synergizes Combined Ultrasound-Stimulated Microbubble and Radiation Therapy in Human Colon Cancer Xenografts
Source: PLoS One. 2014 Apr 15;9(4):e93888. doi: 10.1371/journal.pone.0093888 (PMC3988033; doi:10.1371/journal.pone.0093888)
Supplement: Table S1 — P-value summary for all quantified 24 hours ISEL staining from all treatment conditions. (DOCX) [file pone.0093888.s004.docx]

| **ISEL - 24 Hours** | **Ctrl** | **XRT** | **Dll4 mAb** | **XRT + Dll4 mAb** | **XRT + USMB** | **XRT + USMB + Dll4 mAb** |
| --- | --- | --- | --- | --- | --- | --- |
| **Ctrl** | - | 0.8 | 0.5 | 0.095 | 0.54 | 0.04* |
| **XRT** | - | - | 0.2948 | 0.0937 | 0.0593 | 0.2286 |
| **Dll4 mAb** | - | - | - | 0.3095 | 0.8413 | 0.0519 |
| **XRT + Dll4 mAb** | - | - | - | - | 0.4633 | 0.7832 |
| **XRT + USMB** | - | - | - | - | - | 0.0519 |
| **XRT + USMB + Dll4 mAb** | - | - | - | - | - | - |
