# Supplementary material for: Dll4-Notch Signalling Blockade Synergizes Combined Ultrasound-Stimulated Microbubble and Radiation Therapy in Human Colon Cancer Xenografts
Source: PLoS One. 2014 Apr 15;9(4):e93888. doi: 10.1371/journal.pone.0093888 (PMC3988033; doi:10.1371/journal.pone.0093888)
Supplement: Table S2 — P-value summary for all quantified 7 days ISEL staining from all treatment conditions. (DOCX) [file pone.0093888.s005.docx]

| **ISEL – 7 Days** | **Ctrl** | **XRT** | **Dll4 mAb** | **XRT + Dll4 mAb** | **XRT + USMB** | **XRT + USMB + Dll4 mAb** |
| --- | --- | --- | --- | --- | --- | --- |
| **Ctrl** | - | 0.6 | 0.057 | .028* | 0.7 | 0.028* |
| **XRT** | - | - | 0.8248 | 0.2286 | 0.5066 | 0.0102* |
| **Dll4 mAb** | - | - | - | 0.4 | 0.1 | 0.2845 |
| **XRT + Dll4 mAb** | - | - | - | - | 0.0571 | 0.6857 |
| **XRT + USMB** | - | - | - | - | - | 0.0571 |
| **XRT + USMB + Dll4 mAb** | - | - | - | - | - | - |
