# Supplementary material for: Dll4-Notch Signalling Blockade Synergizes Combined Ultrasound-Stimulated Microbubble and Radiation Therapy in Human Colon Cancer Xenografts
Source: PLoS One. 2014 Apr 15;9(4):e93888. doi: 10.1371/journal.pone.0093888 (PMC3988033; doi:10.1371/journal.pone.0093888)
Supplement: Table S3 — P-value summary for all quantified growth delay from all treatment conditions. (DOCX) [file pone.0093888.s006.docx]

| **Growth Delay** | **Ctrl** | **XRT** | **Dll4 mAb** | **XRT + Dll4 mAb** | **XRT + USMB** | **XRT + USMB + Dll4 mAb** |
| --- | --- | --- | --- | --- | --- | --- |
| **Ctrl** | - | 0.14 | 0.051 | .0091* | 0.1 | 0.0021* |
| **XRT** | - | - | 0.3398 | 0.0102* | 0.8521 | 0.005* |
| **Dll4 mAb** | - | - | - | 0.0439* | 0.4601 | 0.0054* |
| **XRT + Dll4 mAb** | - | - | - | - | 0.007* | 0.038* |
| **XRT + USMB** | - | - | - | - | - | 0.003* |
| **XRT + USMB + Dll4 mAb** | - | - | - | - | - | - |
