# Supplementary material for: Dll4-Notch Signalling Blockade Synergizes Combined Ultrasound-Stimulated Microbubble and Radiation Therapy in Human Colon Cancer Xenografts
Source: PLoS One. 2014 Apr 15;9(4):e93888. doi: 10.1371/journal.pone.0093888 (PMC3988033; doi:10.1371/journal.pone.0093888)
Supplement: Table S4 — P-value summary for all quantified 24 hours VI values from all treatment conditions. (DOCX) [file pone.0093888.s007.docx]

| **VI - 24 Hours** | **Ctrl** | **XRT** | **Dll4 mAb** | **XRT + Dll4 mAb** | **XRT + USMB** | **XRT + USMB + Dll4 mAb** |
| --- | --- | --- | --- | --- | --- | --- |
| **Ctrl** | - | 0.17 | .045* | .0024* | .048* | .0009* |
| **XRT** | - | - | 0.0931 | 0.0087* | 0.3277 | 0.0027* |
| **Dll4 mAb** | - | - | - | 0.0931 | 0.3277 | 0.02* |
| **XRT + Dll4 mAb** | - | - | - | - | 0.012* | 0.75 |
| **XRT + USMB** | - | - | - | - | - | 0.0206* |
| **XRT + USMB + Dll4 mAb** | - | - | - | - | - | - |
