# Supplementary material for: Dll4-Notch Signalling Blockade Synergizes Combined Ultrasound-Stimulated Microbubble and Radiation Therapy in Human Colon Cancer Xenografts
Source: PLoS One. 2014 Apr 15;9(4):e93888. doi: 10.1371/journal.pone.0093888 (PMC3988033; doi:10.1371/journal.pone.0093888)
Supplement: Table S5 — P-value summary for all quantified 7 days VI value from all treatment conditions. (DOCX) [file pone.0093888.s008.docx]

| **VI – 7 Days** | **Ctrl** | **XRT** | **Dll4 mAb** | **XRT + Dll4 mAb** | **XRT + USMB** | **XRT + USMB + Dll4 mAb** |
| --- | --- | --- | --- | --- | --- | --- |
| **Ctrl** | - | 0.5 | .0007* | 0.0732 | 0.96 | .0002* |
| **XRT** | - | - | 0.0381* | 0.1111 | 0.7105 | 0.0283* |
| **Dll4 mAb** | - | - | - | 0.6623 | 0.0008* | 0.7546 |
| **XRT + Dll4 mAb** | - | - | - | - | 0.042* | 0.94 |
| **XRT + USMB** | - | - | - | - | - | 0.0003* |
| **XRT + USMB + Dll4 mAb** | - | - | - | - | - | - |
