# Supplementary material for: Dll4-Notch Signalling Blockade Synergizes Combined Ultrasound-Stimulated Microbubble and Radiation Therapy in Human Colon Cancer Xenografts
Source: PLoS One. 2014 Apr 15;9(4):e93888. doi: 10.1371/journal.pone.0093888 (PMC3988033; doi:10.1371/journal.pone.0093888)
Supplement: Table S6 — P-value summary for all quantified 24 hours CD31 staining from all treatment conditions. (DOCX) [file pone.0093888.s009.docx]

| **CD31 – 24 Hours** | **Ctrl** | **XRT** | **Dll4 mAb** | **XRT + Dll4 mAb** | **XRT + USMB** | **XRT + USMB + Dll4 mAb** |
| --- | --- | --- | --- | --- | --- | --- |
| **Ctrl** | - | 0.5 | .0007* | 0.0732 | 0.96 | .0002* |
| **XRT** | - | - | 0.0952 | 0.2857 | 0.0173* | 0.0519 |
| **Dll4 mAb** | - | - | - | 0.0317* | 0.0087* | 0.0087* |
| **XRT + Dll4 mAb** | - | - | - | - | 0.0667 | 0.2571 |
| **XRT + USMB** | - | - | - | - | - | 0.1797 |
| **XRT + USMB + Dll4 mAb** | - | - | - | - | - | - |
